# Validity and reliability of RPE as a measure of intensity during isometric wall squat exercise

**Published:** 2021-03-24

**Authors:** John W. D. Lea, Jamie M. O’Driscoll, Damian A. Coleman, Jonathan D. Wiles

**Affiliations:** School of Human and Life Sciences, Canterbury Christ Church University, Canterbury, United Kingdom

**Keywords:** Effort, External load, Perceived exertion, Resistance exercise, Workload

## Abstract

**Background and aims:**

Isometric exercise (IE), including wall squat training, has been shown to be effective at reducing resting blood pressure (BP). Rating of perceived exertion (RPE) is also widely used as an accessible additional measure of IE intensity. Despite this, no RPE scales have been specifically designed for use with IE and it is not clear whether RPE is sensitive enough to distinguish between different lower limb IE workloads. Therefore, the aims of this study were to assess the validity and reliability of RPE as a measure of IE intensity (workload) and physiological exertion (Heart rate and BP), and to examine whether RPE is able to discern differences in wall squat workload (knee angle) at a resolution of 10-degrees, as was previous shown for heart rate (HR) and BP.

**Methods:**

Twenty-nine male participants completed eight separate isometric wall squat testing sessions, separated by a minimum of 5-h. Each session consisted of a single 2-min isometric wall squat test, at one of five randomized workloads (knee joint angles). Three of the knee angles were repeated, a second time, to allow measurements of reliability. Throughout the exercise protocol, HR and BP were recorded continuously; values for each 30-s time-point were calculated as the mean of the proceeding 5-s, and peak values for the 2-min bout were taken as the mean results for the final 5-s of the bout. In addition, mean results for the full 2-min period were calculated. RPE was collected every 30 s. Concurrent validity was assessed by correlating RPE results with the criterion measures: Knee joint angle, HR, and BP. Differences in RPE were assessed across consecutive workloads and time-points.

**Results:**

There were significant increases in RPE at each consecutive wall squat workload (P<0.001) and between each consecutive 30-s time point (P<0.001). In addition, the RPE results produced a significant inverse relationship with knee angle (r=−0.79; P<0.001) and significant positive relationships with HR (r=0.53, P<0.001) and BP (systolic: r=0.77; diastolic: r=0.62; and mean arterial pressure: r=0.70, P<0.001).

**Conclusion:**

RPE provides a valid and reliable measure of isometric wall squat intensity, physiological exertion and can discern between knee angles with a resolution of 10°.

**Relevance for patients:**

Patients and practitioners implementing isometric exercise training for arterial blood pressure reduction can use RPE to accurately monitor the intensity of the exercise and the physiological responses.

## 1. Introduction

Cardiovascular disease (CVD) is the leading preventable cause of morbidity and mortality worldwide [1]. Hypertension, characterized by a sustained elevation in arterial blood pressure (≥140 mmHg systolic and/or ≥90 mmHg diastolic), is the leading attributable risk factor for increases in CVD mortality [2]. Exercise has been recommended as a non-pharmacological lifestyle modification for the treatment of hypertension [3]. In recent years, isometric exercise (IE) interventions have been shown to be a time efficient means of reducing resting [4-9] and ambulatory arterial blood pressure [9], with clinically meaningful reductions shown in systolic blood pressure (SBP), diastolic blood pressure (DBP), and mean arterial pressure [MAP] [10-12].

The control of exercise intensity is vital to ensure the safety and efficacy of physical activity in athletic, recreational, or therapeutic settings [13,14]. During IE, the intensity of the exercise can be manipulated by changing the workload (i.e., force), time under tension (i.e., number of contractions and contraction time), or recovery time between efforts. The previous methods of administering and monitoring IE intensities have tended to require expensive equipment such as isokinetic dynamometers [4-6], handgrip dynamometers [15], and electromyography [EMG] [8]. It has been suggested that the need for expensive equipment, as listed previously and time-consuming testing protocols present a potential barrier that will limit the effectiveness of IE interventions [16]. Consequently, more accessible modes of IE that can be implemented in the home have been explored.

One such intervention is the use of isometric wall squat (IWS) training, where workload is manipulated using knee joint angle [17]. These authors demonstrated that HR and BP responses could be reliably altered during a single 2-min bout, with significant increases in physiological parameters detectable with 10-degree decreases in knee joint angle. This study also demonstrated that there were significant increases in HR and BP with increases in time under tension (TUT) at a set workload. Subsequently, a laboratory-based maximal IWS test was developed, utilizing the 10-degree workload resolution, to allow measurement of peak HR and for the relationship between HR and workload to be plotted [18]. This information is then used to calculate a training knee joint angle that will elicit a 95% HR peak training stimulus. Four-week home-based IWS interventions, utilizing this prescription method, have induced significant reductions in resting [9,19] and ambulatory arterial BP [9].

While this methodology has removed several of the barriers to IET intervention, such as the need for expensive equipment and the need to attend the laboratory for each training session, this mode of IET still requires a laboratory-based maximal exercise test and specialist equipment to control knee joint angle in the home. Therefore, if a method of prescribing and monitoring IE could be developed that did not require the measurement of HR or knee angle but still allowed precise control of intensity, these barriers to participation could also be removed.

Rating of perceived exertion (RPE) is widely used as a measure of exercise intensity for many types of exercise testing and training [20], including during IWS training interventions [18]. In addition, RPE has been shown to be a valid measure of exercise intensity and physiological exertion during various forms of resistance exercise including dynamic squats [21], isometric handgrip training [22], and eccentric elbow flexor contractions [23]. Evidence suggests that RPE is accurate during both estimation tasks, where exercise intensity is set and RPE is then estimated by the participant [22,24,25], and production tasks, where the participant is asked to adjust the intensity of the exercise to produce a particular RPE value [22,26,27]. RPE validity has also been suggested to be independent of participant sex [28-30] and age [31,32]. Therefore, RPE could provide a promising alternative method of controlling IWS intensity [22,33]. However, there is little research exploring the implementation of exercise interventions with EI prescribed using RPE. In addition, before RPE can be used to replace HR in this way, it is important to validate RPE for the specific exercise type and modality being used [34]. Indeed, it has been suggested that caution should be taken when using RPE scales with modalities and materials other than those they have been validated for [35]. Furthermore, for an RPE scale to be considered a valid measure for use in the clinical and/or health-fitness setting, it must demonstrate both concurrent and construct validity, evidenced by strong positive correlations with physiological variables (e.g., HR) and a previously validated criterion scale, respectively [36].

The Isometric Exercise Scale (IES) has previously shown excellent construct validity during continuous maximal IWS exercise [37] using the CR-10 scale, which has previously been shown to be valid during isometric exercise [22]. Lea *et al*. [37] also demonstrated the concurrent validity of the IES, using HR and BP, during continuous IE. However, to date, no RPE scale has been validated for use during discontinuous IWS exercise, and it is unknown how precisely RPE can differentiate between different IE workloads or TUT. If the IES can be shown to be a valid and reliable measure of intensity during discontinuous IE, with a resolution sufficient to distinguish workloads within a single 2-min bout, future research could look to replace the current third party administered HR based prescription method with a more participant centered RPE prescription method, where the participant can select their squatting position to produce a specific RPE value.

Therefore, the aims of this research were to: (1) assess the validity of RPE using the Isometric Exercise Scale (IES) as a measure of IWS intensity, during single bouts performed at different angles; (2) examine whether RPE can discern IWS workloads at the same 10-degree resolution as HR and BP; (3) explore the concurrent validity of RPE using the criterion measures of exercise intensity and physiological exertion (HR and BP) during this type of exercise; and (4) examine the reliability of the RPE responses across duplicated sessions.

## 2. Materials and methods

### 2.1. Participants

Twenty-nine males, 17 normotensive and 12 prehypertensive, volunteered to participate in this study (age: 24.3±3.6 years; stature: 180.4±6.8 cm; and body weight: 79.3±14.1 kg). Before testing, written informed consent was obtained from all participants. In addition, participants completed a health and medical questionnaire and self-reported that they were not suffering from any injury or disease. All participants were physically active (6.9±3.5 h of light to moderate exercise per week), non-smokers and not taking any medication during the investigation. Typical habitual exercise included walking, running, cycling, swimming, football, hockey, volleyball, golf, squash, mixed martial arts, and resistance training. Participants agreed to maintain regular dietary and physical activity habits throughout the testing period.

Two a priori sample size calculations were conducted for the primary analyses: the first was for correlation testing of RPE rating with workload, HR, and BP. The second was for repeated measures analysis of variance (ANOVA) to assess differences in mean and peak RPE across the five different workloads. These analyses suggested that *n*=29 and *n*=24 participants were required respectively to obtain a power of 0.8, for a medium effect size with an alpha level of *P*<0.05. Therefore, a minimum sample size of *n*=29 was selected for this study.

### 2.2. Study design

All participants were required to attend the laboratory on eight separate occasions to complete a 2-min wall squat test, at a specific knee joint angle. The wall squat was completed at one of five possible knee joint angles ranging from 135° to 95° in 10° increments, where approximately 180° represents a fully extended knee joint. These angles were chosen as they are currently used in the prescription of IWS exercise [18]. The 125° and 105° angles were completed just once, while the 135°, 115°, and 95° angles were completed twice. These eight sessions were completed in a randomized cross-over design. Participants were not told which knee joint angle they were squatting at and while they were aware that some knee angles would be repeated, they were not told which angles. Sessions were separated by a minimum of 5-h, with a maximum of two testing sessions in any 24-h period. A 5-h rest period has previously been shown to be sufficient to allow full cardiovascular and muscular recovery [17]. Participants were asked to abstain from food 4 h, caffeine 12 h, alcohol, and strenuous exercise 24-h pre-testing. All participants ensured they had an empty bladder and verbally confirmed adherence to the testing requirements before the start of each testing session. This study was approved by the University Ethics Committee (15/SAS/223) and was conducted according to the Declaration of Helsinki.

### 2.3. Procedures

#### 2.3.1. Familiarization

Before all data collection, participants undertook a separate familiarization session, where they were introduced to the testing protocols and all measurement procedures, including the use of the RPE scale.

#### 2.3.2. Resting measures

On arrival at the laboratory participants rested in a seated position for 10 min. After 10 min rest, HR, SBP, DBP, and MAP were recorded using an oscillometric BP monitor on the participants left arm. The cuff was secured flat to the arm, with the midline of the bladder aligned with the brachial artery and the lower end of the cuff positioned 2-3 cm above the antecubital fossa. Three measurements were taken, each separated by 1-min [38]. The average of the seated BP measurements was used to classify participant BP status. Following the seated measurements, participants rested in a supine position for 15 min. After an initial 10-min period, HR and BP (SBP, DBP, and MAP) were measured continuously for 5 min using a plethysmographic device (Task Force^®^ Monitor). Resting HR and BP values were calculated as the mean of the 5-min supine measurement period, and were used to test the between session variance in resting measures.

#### 2.3.3. The isometric wall squat exercise protocol

**Figure 1:**
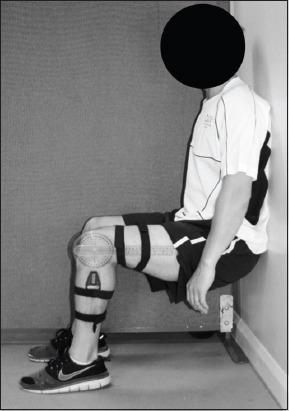
Isometric wall squat position and knee angle measurement using a clinical goniometer with spirit level to ensure vertical position of the lower leg

After completing the initial resting measures, participants performed a 2-min wall squat, at one of the randomly assigned knee joint angles. Participants started upright with their back against the wall, feet parallel and shoulder-width apart, and hands by their side. Participants then lowered their back down the wall whilst moving their feet forward into the required position, determined by the knee joint angle. Knee joint angle was measured using a clinical goniometer (MIE Medical Research, Leeds, UK), secured to the participants lower and upper leg using elasticated Velcro strapping (Figure 1). The fulcrum was aligned with the lateral epicondyle of the femur; the moving arm was placed on the lateral midline of the femur using the greater trochanter for reference and the stationary arm on the lateral midline of the fibula using the lateral malleolus and fibular head for reference. A spirit level was attached to the stationary arm to ensure that the lower leg was kept vertical during the exercise (Figure 1). The internal angle between the femur and fibula was measured. Participants were instructed to keep their lower legs vertical and their trunk erect. Participants were encouraged to complete the wall squat barefoot or to ensure the same non-slip footwear was worn for all testing sessions. The wall squat was held for 2 min or until the point of fatigue. Verbal encouragement was given, and participants were informed of the elapsed time. To avoid the Valsalva maneuver, participants were instructed to breathe normally. Beat-to-beat measurements of HR and BP (SBP, DBP, and MAP) were recorded continuously during the 2-min bout. Mean results were calculated for each variable for the full 2-min period. In addition, to allow comparison of different time-points, results were calculated for each 30 s period (30s, 60s, 90s, and 120s) as the mean of the proceeding 5 s [17]. The mean results for the past 5 s of the final time-point (90-120 s) were taken as the peak results for that test.

#### 2.3.4. Ratings of perceived exertion

**Figure 2:**
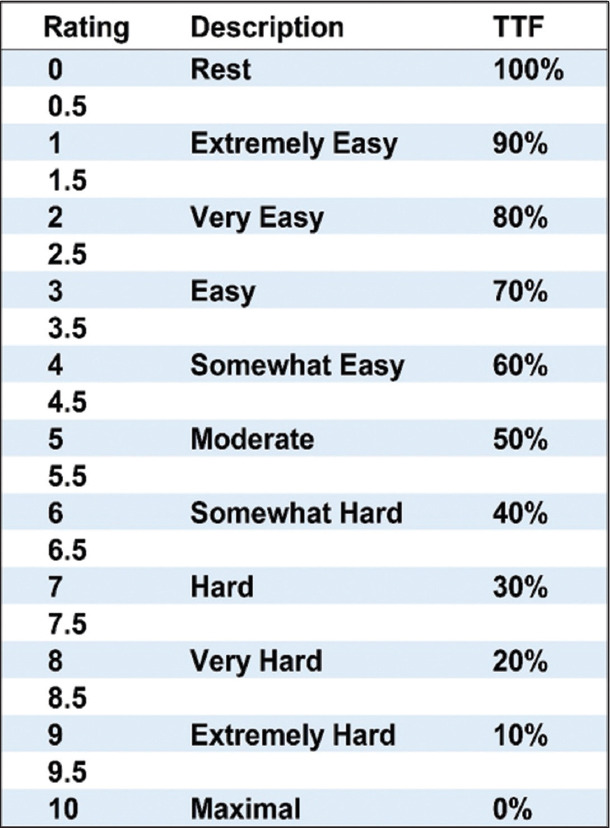
The isometric exercise scale

Participants were asked to rate the perceived exertion in their active muscles, 5 s before the end of each 30 s period of the test. RPE was recorded using the IES (Figure 2), which has previously been shown to be a valid and reliable measure during continuous wall squat exercise [37] Participants were cued to give their ratings using the standardized question, “How hard do you feel your muscles are working?” The scale was positioned in full view of the participant for the entire test. Standardized scaling and anchoring instructions were given to participate before each test, as follows:

“*This scale is used to rate how hard you think your active muscles are working. This scale has three different columns: Rating, Description and TTF. The “Rating” numbers are from 0-10 and are used to rate the exertion or effort in the active muscle group(s). The “Description” words and “TTF” are used to help you choose a rating. 0 (Rest) is absolutely no effort, as felt during complete rest. 5 (Moderate) is right in the middle of 0 and 10. Its not especially hard and it is no problem to continue; but it no longer feels comfortable. 10 (Maximal) is maximum effort; your muscles are working as hard as they can*, and *you can only maintain this for seconds before you will have to stop. TTF (Time to Failure) indicates the amount of time remaining, during an isometric contraction, before you will be unable to continue. In other words, this describes how much you have left in your ‘fuel tank’. 100% - your muscles are fresh; you haven’t started the contraction yet (fuel tank is full). 50% - means you can continue to hold the contraction for the same amount of time that you have already completed (fuel tank is half full). 0% - your muscles are failing/have failed (fuel tank is empty). When you give your rating; focus only on the muscle group(s) that is working. You can use the “Description” words, the Time to Failure (TTF), and/or you can simply rate the exertion out of ten.”*

### 2.4. Data analysis

All data were analyzed using the SPSS (Version 24; SPSS Inc., Chicago IL). Before analysis, all data were checked for conformity with the parametric assumptions [39].

The mean resting HR and BP results, collected at the start of each session, were assessed for statistical differences between the eight sessions using either one-way repeated-measures ANOVA or Friedman’s related-samples analysis of variance, normal distribution dependent.

The concurrent validity of RPE with the criterion measure of exercise intensity, Knee Joint Angle, and the criterion measures of exercise intensity and physiological exertion, HR, and BP, was assessed using Spearman’s rank-order correlations. In addition, the relationships between workload and the physiological variables were measured using Pearson’s product-moment correlations (HR and SBP) and Spearman’s rank-order correlations (DBP and MAP).

Mean RPE results were assessed for differences between workloads using a Friedman’s related-samples analysis of variance test, with Wilcoxon signed-rank tests for *post hoc* comparisons. For peak results across each workload and time point, HR was assessed using a two-factor (workload x time) repeated measures analysis of variance (ANOVA) with *post hoc* paired-samples t-tests, while for RPE and BP, non-parametric Friedman’s tests were used with the Wilcoxon signed-rank tests for *post hoc* comparisons. All *post hoc* testing used a Bonferroni adjustment for multiple comparisons.

Reliability of the peak IES, HR, and BP results between the repeated sessions were examined separately using: (1) two-factor (session x workload) repeated measures ANOVA’s or Friedman’s test. *Post hoc* testing was conducted using dependent *t*-tests or Wilcoxon signed-rank test’s (normal distribution dependent), with Bonferroni adjustment for multiple comparisons; (2) Intraclass Correlation Coefficients (ICC) and Standard Error of Measurements (SEM) were calculated between the repeated session, for each variable, to assess agreement between the repeated measures. An alpha level of <0.05 was set as the threshold for statistical significance. All data are expressed as mean±S.D., unless otherwise indicated.

## 3. Results

### 3.1. Resting measures

At the start of each of the eight testing sessions resting measures were recorded for each participant. The mean seated BP measurements were: SBP: 119±7 mmHg; DBP: 68±7 mmHg; MAP 87±6 mmHg, with 17 participants classified as normotensive and 12 classified as prehypertensive. The mean supine resting values for HR, SBP, DBP, and MAP were: 61±7 beats min^−1^, 113±10 mmHg, 66±8 mmHg, and 84±8 mmHg, respectively. There were no significant differences in any resting measures between trials (*P* > 0.05). The ICC results for the supine resting measures ranged from 0.71 to 0.80.

### 3.2. Exercising measures

**Figure 3:**
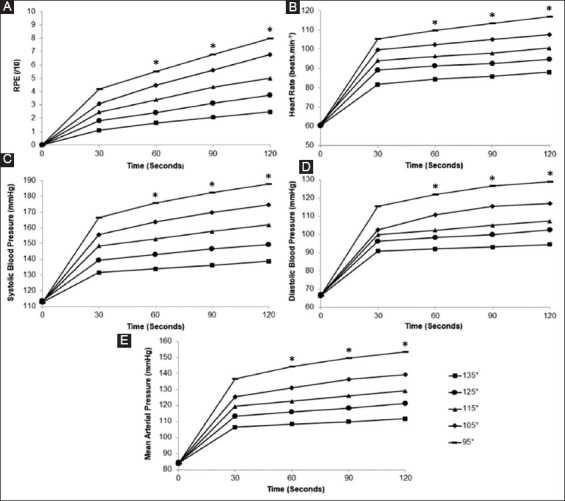
(A-E) RPE, heart rate, and blood pressure results for each 30-s time point throughout the isometric contraction. HR and BP results are the mean for the last 5-s of that time point. *indicates significant increase in results, for all knee joint angles, when compared to the previous time-point. Legend – angles represent the internal knee joint angle.

All the participants completed each knee joint angle for the full 2-min. Mean (the average of all measurements taken during the 2-min IE bout) and peak (the mean of the past 5-s of each 2-min IE bout) results were calculated for RPE, HR, and BP for each knee joint angle; the peak results were significantly higher (*P*<0.001) than the mean results for all variables at all workloads. Significant increases in mean RPE results (Table 1) were seen with each consecutive increase in workload [F(1, 28)=337.29, *P*<0.001). A significant interaction between the effects of workload and time-points on RPE [X^2^(19)=501.07, *P*<0.001]. *Post hoc* testing revealed significant increases in peak RPE results (Table 2) with each consecutive squatting workload (*P*<0.001) and 30-s time point (*P*<0.001; Figure 3A and B). Likewise, significant increases in HR and BP were seen for all consecutive squatting workloads (*P*<0.001) and time points (*P*<0.001), for each BP parameter (Figure 3C-E).

**Table 1:** Mean RPE, heart rate, and blood pressure results at each knee joint angle

	Knee joint angle				

	135°	125°*	115°*	105°*	95°*
RPE (/10)	1.8±1.2	2.8±1.3	3.8±1.2	5.0±1.4	6.1±1.4
HR (beats.min^−1^)	85±11	92±11	97±10	104±10	111±11
SBP (mmHg)	135±15	145±14	155±14	166±13	178±17
DBP (mmHg)	93±13	99±14	104±13	111±15	123±20
MAP (mmHg)	109±14	117±13	124±14	133±13	146±18

* Indicates a significant increase in all variables when compared to the previous (higher) knee joint angle

**Table 2:** Peak RPE, heart rate, and blood pressure values for each 2-min bout including repeat reliability sessions

	Knee joint angle							

	135°^a^	135°^b^	125°	115°^a^	115°^b^	105°	95°^a^	95°^b^
RPE (/10)	2.3±1.6	2.3±1.5	3.7±1.6	4.9±1.6	5.1±1.6	6.8±1.8	8.1±1.7	7.9±1.7
HR (b.min^−1^)	88±12	88±11	95±11	100±11	101±10	108±10	116±12	117±11
SBP (mmHg)	135±16	139±15	149±14	162±14	162±14	175±14	188±18	188±21
DBP (mmHg)	92±14	92±12	102±13	107±14	107±14	117±17	129±22	129±24
MAP (mmHg)	109±14	112±13	121±13	130±15	129±14	139±13	153±19	154±22

Peak values calculated for the last 5-s of each bout. (a) first session completed at that workload; (b) repeat session at that workload

### 3.3. Exercise measures and knee angle

**Figure 4:**
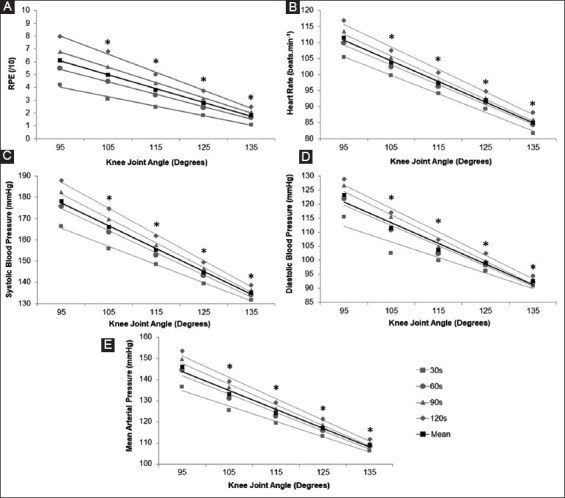
(A-E) The effect of knee joint angle on RPE, heart rate, and blood pressure. Black lines are the mean results for each 2-min contraction. Grey lines display results for each 30-s time-point at a given workload. *Indicates a significant reduction in the mean result when compared to the previous (lower) knee joint angle.

Ratings of perceived exertion and knee joint angle produced a significant inverse relationship (*r*=−0.78, *P*<0.001; Figure 4A). Likewise, a significant inverse relationship was found between HR and knee joint angle (*r*=−0.68, *P*<0.001; Figure 4B). The correlations between BP parameters (SBP, DBP, and MAP) and knee joint angle also revealed significant inverse relationships (*r*=−0.79, *r*=−0.62 and *r*=−0.72, respectively; *P*<0.001), Figure 4.

### 3.4. RPE and measures of physiological exertion

Exploration of the relationships between RPE and the concurrent measures of exercise intensity: HR, SBP, DBP, and MAP, produced significant positive correlations of *r*=0.53, *r*=0.77, *r*=0.62, and *r*=0.70 (*P*<0.001), respectively.

### 3.5. Reliability of exercise measures

Reliability of the peak values for each exercise variable (RPE, HR, and BP) was assessed between the first and second training sessions at the 135°, 115°, and 95° workloads (Table 2). There were no significant differences between trials (*P* > 0.05), for any of the exercise variables at any workload. The ICC result (with 95% confidence intervals) for RPE was 0.78 (0.65-0.88) with SEM of 0.65. Heart rate showed excellent between session agreement, ICC 0.96 (0.92-0.98) and SEM=2.4. Likewise, SBP (ICC 0.95, 0.91-0.97; SEM=3.4), DBP (ICC 0.72, 0.72-0.91; SEM=6.5), and MAP (ICC 0.92, 0.86-0.96; SEM=4.5) showed excellent between session reliability.

## 4. Discussion

This study demonstrated that RPE is a valid measure of exercise intensity during discontinuous IWS exercise; as shown by the strong positive validity coefficients between both mean and peak RPE ratings and workload, which exceeded those shown previously during dynamic squatting exercise [25]. In this study, the measurement of IE intensity (i.e., how hard the isometric hold is or was) was split into two main factors: workload (knee joint angle) and TUT; to inform future research looking to prescribe IE using RPE, it was important to assess the resolution at which RPE can discern between changes in exercise intensity caused by manipulation of both these variables during a single 2-min bout. The RPE results increased significantly (*P*<0.05) with each consecutive time point and workload. Thus, RPE was sensitive enough to distinguish between 10-degree increments in knee joint angle and TUT at each 30-s time point. This same resolution has previously been shown to be the limit for HR and BP, during single bouts of IWS exercise [17]. The results of the current study show that RPE is sufficiently accurate to distinguish wall squat workloads within a 2-min period, which is the length of each contraction in the current IWS protocol for BP reduction [19]. Meaning it would be possible to use RPE to monitor and adjust workload within the very first contraction of each training session.

The RPE results produced strong positive relationships with HR and BP results. These validity coefficients are in line with those previously shown during dynamic squatting [21] and isometric handgrip exercise [22], and suggest that RPE can accurately represent the changes in physiological exertion during this mode of IE. In addition, the HR and BP results from this research showed the same 10-degree knee joint angle and 30-s TUT resolution as was previously shown by Goldring *et al*. [17] and by the RPE results in the current study. These results suggest that RPE is valid measure of physiological exertion, and is able to monitor and reflect changes in isometric EI with the same accuracy as HR and BP. As such, this research shows that RPE is a useful measure for monitoring IWS intensity and lays the foundations for future studies to investigate the use of RPE to replace HR in the prescription of IE interventions.

The ICC results showed excellent agreement for the RPE ratings given in the repeated sessions (*r*=0.78; 0.65-0.88). This result closely matches the findings of previous studies that have shown good RPE reliability, using different RPE scales and resistance exercise modalities; such as during explosive resistance exercise using Borg’s 6-20 scale [*r*=0.729] [40], using a modified OMNI-Res scale during elastic band exercise [*r*=0.76] [41], and using the Thera-band resistance exercise scale during elastic band exercise [*r*=0.67] [34]. The ICC results for the physiological variables also showed excellent agreement between sessions. To reduce the burden placed on participants and consequently attrition rate, only three of the knee joint angles (135°, 115°, and 95°) were repeated as a measure of reliability. While it may have been preferable to conduct reliability testing at each knee joint angle, the current sample represented the highest, lowest, and middle workloads used in the current selection protocols for IWS interventions [19], and therefore results would suggest that RPE is reliable between sessions across the full range of wall squat workloads required for prescription and monitoring of this mode of exercise.

One further limitation of this research was the homogeneous sample of young male participant used. This study had a maximum age for participation of 65-years; however, the convenience sample used and the university setting for the study lead to a sample of younger males. Females were excluded from this study, as the menstrual cycle has been shown to affect BP readings [42,43] and as this study was conducted over a period of up to 1-month it was guaranteed that measurements would be taken at different stages of the cycle. Future research should examine the accuracy and resolution of RPE in older populations and female populations of all ages.

Goldring *et al*. [17] showed that manipulation and monitoring of knee angle based on HR and BP provided a valid way to produce, select, and monitor an isometric training stimulus. This has subsequently been used to carry out isometric training interventions sufficient to cause reductions in resting and ambulatory BP in just 4 weeks [9]. This current research has shown that RPE can provide an equally valid and reliable measure of exercise intensity and workload during the first 2-min of this exercise. Therefore, RPE could be used to monitor changes in exercise intensity during IE training sessions and could also be used to develop a new IE prescription protocol that would remove the need for laboratory visits and maximal exercise tests, which may remove possible barriers to participation in people who could otherwise benefit from this type of intervention.

Further research is required to explore whether the validity and reliability of RPE remains strong during multiple 2-min wall squat bouts, separated by 2-min rest, in a home-based setting, as is currently used in interventions to reduce arterial BP [19]. In addition, research could examine whether RPE can be used to accurately prescribe IWS intensities, as was shown in isometric handgrip exercise by Morrin *et al*. [22]; and whether RPE prescribed exercise can reliably produce HR and BP levels sufficient to cause a reduction in resting BP. Finally, the efficacy of self-administered RPE, using the standard instructions and anchoring, should be explored to allow IE training with no intervention from an exercise professional or researcher.

## 5. Conclusion

Ratings of perceived exertions from the IES scale provide a valid and reliable measure of IWS intensity. RPE ratings are sufficiently accurate to distinguish between knee joint angles at a 10° increment, as was previously shown for HR and BP. In addition, changes in perceived exertion accurately represented the changes in physiological exertion, measured using HR and BP. As such, RPE is sufficiently accurate to be useful for monitoring IE intensity, and could be used to develop an alternative prescription method for IWS interventions to provide a sufficient training stimulus to reduce resting and ambulatory BP, while removing the need for expensive equipment and maximal testing.
